# Prognostic Value of Heart‐Type Fatty Acid‐Binding Protein in Patients With Acute Coronary Syndrome: A Systematic Review and Meta‐Analysis

**DOI:** 10.1155/cdr/4836688

**Published:** 2026-10-20

**Authors:** Ahmed Elmewafy, Eyad Jamileh, Abdullah Hussain, Loay Kamel, Omar Farooq, Hany Gamal, Ibrahim Antoun

**Affiliations:** ^1^ School of Medicine University of Sheffield Sheffield UK sheffield.ac.uk; ^2^ Royal Blackburn Teaching Hospital East Lancashire Hospitals NHS Trust Blackburn UK nhs.uk; ^3^ School of Medicine Royal College of Surgeons in Ireland Medical University of Bahrain Busaiteen Kingdom of Bahrain uob.edu.bh; ^4^ School of Medicine Royal College of Surgeons of Ireland University of Medicine and Health Sciences Dublin Ireland rcsi.ie; ^5^ School of Medicine St. George′s University St. George′s Grenada; ^6^ Division of Cardiovascular Sciences Clinical Science Wing University of Leicester Glenfield Hospital Leicester UK nhs.uk

**Keywords:** acute coronary syndrome, biomarkers, heart-type fatty acid-binding protein, meta-analysis, prognosis

## Abstract

**Introduction:**

Heart‐type fatty acid‐binding protein (H‐FABP) is rapidly released following myocardial injury, but its prognostic significance in acute coronary syndrome (ACS) remains uncertain. We evaluated the association between elevated H‐FABP and adverse clinical outcomes in confirmed or suspected ACS.

**Methods:**

PubMed, Embase, CENTRAL, Web of Science and Scopus were searched from inception to 30 August 2026 without date restriction. Studies assessing diagnostic and/or prognostic relevance of circulating H‐FABP in adults with confirmed or suspected ACS were eligible. Prognostic outcomes included all‐cause mortality, major adverse cardiovascular events (MACE), heart failure (HF)–related hospitalisation and recurrent myocardial infarction (MI). Risk of bias was assessed using QUIPS. Crude odds ratios (ORs) from extractable 2 × 2 event data were pooled using inverse‐variance random‐effects models with restricted maximum‐likelihood estimation.

**Results:**

Twelve studies were included, with five contributing to meta‐analysis. Elevated H‐FABP was associated with higher odds of all‐cause mortality (OR 5.68; 95% CI 3.98–8.09; *I* ^2^ = 30*%*), MACE (OR 4.56; 95% CI 1.97–10.55; *I* ^2^ = 47*%*), HF‐related hospitalisation (OR 4.29; 95% CI 2.94–6.26; *I* ^2^ = 0*%*) and recurrent MI (OR 1.65; 95% CI 1.15–2.37; *I* ^2^ = 0*%*). Pooled estimates were unadjusted; seven studies were synthesised narratively.

**Conclusion:**

Elevated H‐FABP is associated with adverse outcomes in ACS, but current evidence does not establish independent prognostic value. Further prospective studies should determine whether H‐FABP adds predictive value beyond established clinical and biochemical risk markers.

## 1. Introduction

Acute coronary syndrome (ACS), encompassing unstable angina, ST‐elevation myocardial infarction (STEMI) and non–ST‐elevation acute coronary syndrome (NSTE‐ACS), remains a major contributor to cardiovascular morbidity and mortality worldwide [1]. Accurate diagnosis and risk stratification are therefore central to the early management of patients presenting with suspected ACS. Cardiac troponins remain the principal biomarkers for detecting myocardial injury and are integral to contemporary diagnostic pathways [2].

Heart‐type fatty acid‐binding protein (H‐FABP) is a low‐molecular‐weight cytoplasmic protein involved in intracellular fatty‐acid transport in cardiomyocytes [3]. Because of its small molecular size and cytoplasmic location, H‐FABP is released rapidly into the circulation following myocardial injury [4–6]. Concentrations may rise during the first hours after symptom onset, peak within several hours and subsequently decline rapidly, partly because of renal elimination [5, 6]. These characteristics initially generated interest in H‐FABP as an early biomarker of myocardial injury.

Beyond diagnosis, H‐FABP has also been investigated as a marker of prognosis. Individual studies have reported associations between elevated H‐FABP and mortality, recurrent myocardial infarction, heart failure and composite cardiovascular outcomes [4]. However, differences in ACS populations, assay platforms, H‐FABP thresholds, sampling times, renal‐function handling, follow‐up periods and outcome definitions have complicated interpretation of the evidence.

The most recent dedicated systematic review by Jones et al. [7] was published in 2017 and synthesised evidence available to mid‐2015 without undertaking a quantitative meta‐analysis. An updated synthesis is therefore warranted. Interest in prognostic biomarkers in ACS also continues beyond markers of myocardial necrosis. For example, Karakayali et al. [8] recently reported that serum malondialdehyde, an oxidative‐stress biomarker, was independently associated with in‐hospital mortality in a cohort of 556 patients with ACS. Such studies illustrate continuing interest in biomarkers capable of complementing established clinical risk assessment.

The present systematic review and meta‐analysis therefore aimed at evaluating the association between H‐FABP and clinically relevant outcomes in patients with confirmed or suspected ACS, including MACE, all‐cause mortality, recurrent MI and HF‐related hospitalisation. Diagnostic studies permitted by the registered protocol were retained as contextual narrative evidence where they informed biomarker timing, diagnostic discrimination or early risk stratification. The review additionally sought to characterise important sources of clinical and methodological heterogeneity and identify priorities for future research.

## 2. Methods

### 2.1. Reporting and Registration

This systematic review was conducted and reported in accordance with the Preferred Reporting Items for Systematic Reviews and Meta‐Analyses (PRISMA) 2020 statement [9]. The protocol was prospectively registered with the International Prospective Register of Systematic Reviews (PROSPERO; CRD420251113979) [10]. The completed PRISMA 2020 checklist is provided as File S1.

### 2.2. Study Eligibility

Studies were eligible if they included adults with confirmed or suspected ACS, including STEMI, NSTE‐ACS, unstable angina or patients presenting with acute chest pain or other symptoms in whom ACS was clinically suspected; measured circulating H‐FABP; and evaluated its diagnostic and/or prognostic relationship with clinically relevant outcomes.

H‐FABP exposure was defined according to the threshold, concentration category or qualitative classification used by each original study. Where applicable, patients with elevated or positive H‐FABP were compared with patients with lower, normal or negative H‐FABP.

Observational studies evaluating diagnostic performance, prognostic outcomes or both were eligible in accordance with the registered protocol. No minimum follow‐up duration was imposed because early diagnostic and risk‐stratification outcomes as well as longer term prognostic outcomes were of interest. Eligible prognostic outcomes included all‐cause or cardiovascular mortality, MACE, recurrent MI, HF‐related hospitalisation and other clinically relevant cardiovascular outcomes. Diagnostic studies were retained for narrative synthesis where they evaluated H‐FABP performance in confirmed or suspected ACS and provided relevant evidence concerning biomarker timing, diagnostic discrimination or early clinical risk stratification.

Reviews, editorials, case reports, nonhuman studies and reports from which no relevant diagnostic or prognostic information concerning H‐FABP could be evaluated were excluded. Only English‐language full‐text reports were included. No publication‐date restriction was applied.

### 2.3. Search Strategy

Systematic searches were conducted in PubMed, Embase, the Cochrane Central Register of Controlled Trials (CENTRAL), Web of Science and Scopus from database inception to 30 August 2026, which was the final search date across all databases.

Controlled vocabulary, including Medical Subject Headings (MeSH) and Emtree terms where applicable, and free‐text terms relating to ACS, myocardial infarction, H‐FABP, prognosis, mortality, diagnosis and clinically relevant cardiovascular outcomes were adapted to the syntax and indexing conventions of each database.

No publication‐date restriction was applied at either the database‐search or study‐screening stage. The searches identified 291 records from PubMed, 892 from Embase, 70 from CENTRAL, 254 from Web of Science and 789 from Scopus, yielding 2296 records before deduplication. Complete database‐specific search strategies are provided in File S2.

Records retrieved from all five databases were combined and imported into Rayyan QCRI for automated and manual duplicate removal. Titles and abstracts were screened independently by two reviewers (L.K. and A.H.) using the prespecified eligibility criteria. Full texts of potentially eligible reports were subsequently assessed for inclusion. Disagreements were resolved by consensus or consultation with a third reviewer (A.E.). Reference lists of included studies and relevant systematic reviews were also screened for additional eligible reports.

Articles not published in English were excluded because translation of non‐English full texts was not feasible within the resources available for this review.

### 2.4. Data Extraction

Data were extracted independently by two reviewers (A.E. and A.H.) using a standardised Excel extraction form, with discrepancies resolved by consensus.

Extracted variables included authors, publication year, country, study design, sample size, population characteristics, ACS phenotype and diagnostic status, H‐FABP assay, measurement units, timing of H‐FABP sampling, H‐FABP threshold and basis of threshold where reported, troponin status, renal‐function exclusion criteria or adjustment, follow‐up duration, study‐specific outcome definitions, MACE components, recurrent‐MI definitions, adjusted prognostic estimates and raw 2 × 2 event data where available.

### 2.5. Study Outcomes

The primary prognostic outcomes were all‐cause mortality and MACE stratified by H‐FABP status. Cardiovascular mortality was also extracted where reported. Because the components of MACE differed across studies, MACE was accepted according to the definition used by each original study rather than treated as a uniform composite.

Study‐specific outcome definitions and follow‐up windows were extracted and are reported in Table 1. Secondary prognostic outcomes included recurrent MI, HF‐related hospitalisation and other adverse cardiovascular outcomes.

**Table 1:** Characteristics of included studies, H‐FABP measurement and outcome assessment.

Study	Study design	Population/ACS status	H‐FABP assay	Units	Sampling time	H‐FABP cutoff	Basis of cutoff	Follow‐up/outcome window	Outcome definition	Renal‐function handling	Main findings
Jeong et al. [11]	Observational cohort	278 patients with elevated NT‐proBNP: ACS/cardiac arrest $\begin{array}{c} n=62 \end{array}$, non‐ACS cardiac disease $\begin{array}{c} n=156 \end{array}$, infection/sepsis $\begin{array}{c} n=60 \end{array}$	HiSens H‐FABP LTIA; immunoturbidimetric assay on ADVIA 2400	ng/mL	At enrolment	≥ 8.8 ng/mL for highest category	ROC/AUC‐derived concentration categories	150 days; mortality assessed serially, including short‐term performance to 60 days	Mortality/survival	Cox estimates adjusted for age; renal function not included in reported adjustment	H‐FABP ≥ 8.8 ng/mL was associated with higher mortality; H‐FABP showed strong discrimination for short‐term mortality
Banu et al. [12]	Cross‐sectional observational study	160 confirmed STEMI patients	Randox cardiac Biochip using two monoclonal antibodies	ng/mL	Admission; analysed at < 3 h, 3–6 h and > 6 h after symptom onset	> 6.32 ng/mL	99th‐percentile/reference threshold	In‐hospital	In‐hospital mortality and complications	Renal‐function tests obtained; no multivariable renal adjustment reported	Hs‐cTnT was more sensitive than H‐FABP; H‐FABP was not significantly associated with in‐hospital mortality
Matsumoto et al. [13]	Prospective observational cohort	1283 survivors of confirmed AMI	MARKIT‐M H‐FABP ELISA, DS Pharma Biomedical	ng/mL	Convalescent phase; median 20 days after AMI, range 14–27 days	> 6.08 ng/mL	ROC‐derived optimum for all‐cause mortality	Median 1785 days	All‐cause mortality, nonfatal MI, HF readmission	CKD defined by eGFR < 60 mL/min/1.73 m^2^ included in adjusted outcome analysis	Elevated H‐FABP independently predicted all‐cause mortality and HF readmission but not nonfatal MI
Garcia‐Valdecasas et al. [14]	Prospective observational cohort	165 emergency‐department patients with chest pain < 6 h; suspected AMI/ACS	Dainippon Pharmaceutical ELISA	ng/mL	Blood collected within first 6 h after admission/symptom presentation	6.2 ng/mL	Established diagnostic/reference threshold	6 months	Death, new AMI > 24 h after admission, angina, HF, revascularisation or other cardiac events	Renal failure recorded; no renal variable retained in final reported Cox model	Elevated H‐FABP independently predicted six‐month adverse cardiac events
Kurz et al. [15]	Prospective diagnostic‐accuracy study	94 patients with suspected NSTE‐ACS	Turbidimetric latex immunoassay	*μ*g/L	Presentation with serial samples; early < 4 h versus late ≥ 4 h presenters	ROC‐optimised baseline threshold approximately 3.1 *μ*g/L	Study‐derived ROC threshold	Index presentation/serial diagnostic sampling	Diagnostic identification of myocardial necrosis/NSTEMI	Patients with GFR < 60 mL/min/1.73 m^2^ excluded	H‐FABP demonstrated high early diagnostic sensitivity; hs‐cTnT performed at least as well overall
Viswanathan et al. [16]	Prospective observational cohort	1080 suspected ACS recruited; 955 analysed after exclusion of STEMI/new LBBB and other prespecified exclusions	Randox Evidence Cardiac Biochip chemiluminescent immunometric assay	*μ*g/L	12–24 h after symptom onset	> 6.48 *μ*g/L used to identify higher risk concentrations	Study‐derived prognostic concentration grouping	Minimum 12 months; median 18 months	Death or readmission with MI	Multivariable models incorporated serum creatinine; troponin‐negative analyses adjusted for age and creatinine	H‐FABP independently predicted death or MI and identified high‐risk troponin‐negative patients
McCann et al. [17]	Prospective observational cohort	664 recruited with acute ischaemic‐type chest pain; 555 analysed; 550 with 1‐year follow‐up	Human H‐FABP ELISA, Hycult Biotechnology	ng/mL	Initial/admission sample; median approximately 6 h after chest‐pain onset	≥ 5 ng/mL	Previously published upper limit of normal	1 year	Primary: death or MI; secondary outcomes included death, MI and revascularisation	Serum creatinine/eGFR assessed; eGFR entered into multivariable modelling	Admission H‐FABP provided prognostic information for death or MI independently of established clinical and biomarker variables
Kilcullen et al. [18]	Prospective observational cohort	1448 patients with confirmed ACS	Two‐step direct sandwich ELISA using Dainippon monoclonal antibodies	*μ*g/L	12–24 h after symptom onset	> 5.8 *μ*g/L	99th percentile of 96 healthy controls	12 months	All‐cause mortality	GRACE‐adjusted analyses incorporated admission creatinine	H‐FABP demonstrated a graded independent association with one‐year mortality and added prognostic information to GRACE variables, troponin and hs‐CRP
O′Donoghue et al. [4]	Biomarker substudy of OPUS‐TIMI 16	2287 confirmed ACS patients	Sequential sandwich immunoassay, Biosite Diagnostics	ng/mL	Enrollment; mean approximately 41 ± 20 h after chest‐pain onset	≥ 8 ng/mL	Assay lower detection limit used to define elevation	10 months	Death, recurrent MI, CHF and composite of death/MI/CHF	Creatinine > 1.6 mg/dL or CrCl < 40 mL/min excluded; creatinine clearance included in adjusted model	H‐FABP predicted death, CHF and the composite outcome after multivariable adjustment
Suzuki et al. [19]	Retrospective observational study	90 patients with confirmed ACS	Rapicheck rapid qualitative H‐FABP test, Dainippon	Qualitative positive/negative	As soon as possible after ER presentation	Positive qualitative test	Manufacturer‐defined qualitative positivity	30 days	MACE defined as cardiac death or recurrent ACS	Creatinine > 2.0 mg/dL assessed and entered into Cox analysis	H‐FABP positivity independently predicted 30‐day adverse events
Ishii et al. [20]	Prospective observational cohort	328 consecutive patients hospitalised with ACS within 6 h; 241 had AMI	Two‐step direct sandwich ELISA, MARKIT‐M H‐FABP, Dainippon	*μ*g/L	Admission within 6 h of chest‐pain onset	≥ 9.8 *μ*g/L	Cohort median selected as prognostic cutoff	6 months	Cardiac death or subsequent nonfatal AMI	Patients above prespecified serum‐creatinine threshold excluded; creatinine included in multivariable models	H‐FABP independently predicted six‐month cardiac events, whereas cTnT did not remain independently associated
Nakata et al. [21]	Multicentre prospective observational study	133 patients presenting with suspected ACS; 90 ultimately diagnosed with ACS	Two‐step direct sandwich ELISA using monoclonal antihuman H‐FABP antibodies	ng/mL	First sample within 30 min of ER arrival; repeated at 6‐h intervals for up to 48 h	6.2 ng/mL prespecified; ROC‐optimal threshold 9.5 ng/mL for early ACS detection	Prespecified reference threshold with additional ROC analysis	At least 1 month; key early outcomes within 7 days	Emergency hospitalisation, coronary angiography, revascularisation, recurrent nonfatal MI or death	History of renal failure/renal disease excluded	H‐FABP showed strong early diagnostic performance and high sensitivity for identifying patients requiring urgent hospitalisation, angiography or intervention

Abbreviations: ACS, acute coronary syndrome; AMI, acute myocardial infarction; CHF, congestive heart failure; CKD, chronic kidney disease; CrCl, creatinine clearance; cTnT, cardiac troponin T; eGFR, estimated glomerular filtration rate; ELISA, enzyme‐linked immunosorbent assay; ER, emergency room; GRACE, Global Registry of Acute Coronary Events; HF, heart failure; H‐FABP, heart‐type fatty acid‐binding protein; hs‐CRP, high‐sensitivity C‐reactive protein; hs‐cTnT, high‐sensitivity cardiac troponin T; LBBB, left bundle‐branch block; LTIA, latex turbidimetric immunoassay; MACE, major adverse cardiovascular events; MI, myocardial infarction; NSTE‐ACS, non–ST‐elevation acute coronary syndrome; STEMI, ST‐elevation myocardial infarction.

The definition of recurrent MI, including whether spontaneous and/or periprocedural events were included, was extracted where reported. Where sufficient information was not provided in the original study, this was recorded as not reported.

Diagnostic and early risk‐stratification outcomes from studies eligible under the protocol were synthesised narratively and did not contribute to pooled prognostic effect estimates unless extractable prognostic event data were also reported.

### 2.6. Risk of Bias

Risk of bias was assessed using the Quality in Prognosis Studies (QUIPS) framework across six domains: study participation, study attrition, prognostic‐factor measurement, outcome measurement, study confounding and statistical analysis and reporting.

Each domain was judged as low, moderate or high risk of bias. Two reviewers independently performed assessments, with disagreements resolved by consensus or involvement of a third reviewer. No numerical summary score was calculated, and no separate overall study‐level score was assigned. Risk of bias was interpreted from the pattern of domain‐level judgements, with particular attention to confounding.

Domain‐level assessments are presented in Table 2 and visually summarised in Figure S1.

**Table 2:** Risk of bias assessment using the QUIPS domains.

Study	Study participation	Study attrition	Prognostic‐factor measurement	Outcome measurement	Study confounding	Statistical analysis and reporting
Jeong et al. [11]	Low	Moderate	Low	Low	Low	Low
Banu et al. [12]	Low	Low	Low	Moderate	High	Moderate
Matsumoto et al. [13]	Low	Moderate	Low	Low	Moderate	Low
Garcia‐Valdecasas et al. [14]	High	Low	Moderate	Moderate	High	Moderate
Kurz et al. [15]	High	Low	Moderate	Moderate	Moderate	Moderate
Viswanathan et al. [16]	High	Low	Moderate	Moderate	High	Moderate
McCann et al. [17]	Low	Low	Low	Low	Moderate	Low
Kilcullen et al. [18]	High	Low	Moderate	Moderate	High	Moderate
O′Donoghue et al. [4]	High	Low	Moderate	High	Moderate	Moderate
Suzuki et al. [19]	Moderate	Low	Moderate	Moderate	Low	Moderate
Ishii et al. [20]	High	Low	Moderate	Moderate	High	Moderate
Nakata et al. [21]	Moderate	Low	Moderate	Moderate	Low	Moderate

Abbreviation: QUIPS, Quality in Prognosis Studies.

### 2.7. Data Synthesis

When two or more studies reported extractable 2 × 2 event data stratified by H‐FABP status, we calculated crude ORs and 95% confidence intervals (CIs) from reported event counts and pooled them using inverse‐variance random‐effects models with REML estimation in SPSS Statistics Version 31.0.0.

Adjusted ORs and hazard ratios were not pooled because covariate adjustment, effect measures and reporting were heterogeneous across studies. Adjusted estimates were therefore synthesised narratively where available.

No zero‐event cells requiring continuity correction occurred in the comparisons included in the quantitative synthesis; therefore, no continuity correction was applied.

Follow‐up duration varied substantially between studies. Because sufficiently comparable time‐to‐event estimates were unavailable across studies, binary outcome data were pooled at the follow‐up interval reported by each study. Variation in follow‐up duration was consequently considered an important source of clinical heterogeneity.

Statistical heterogeneity was quantified using the *I* ^2^ statistic. Given that only two to four studies contributed to individual pooled outcomes, *I* ^2^ estimates were interpreted cautiously. Hartung–Knapp adjustment was considered but was not applied; conventional normal‐based CIs were retained.

Formal subgroup analyses, leave‐one‐out analyses and formal publication‐bias testing were not undertaken because of the small number of studies contributing to each pooled outcome.

## 3. Results

### 3.1. Study Selection and Characteristics

The five database searches identified 2296 records before deduplication. Following duplicate removal and title and abstract screening, 20 reports underwent full‐text assessment. Eight reports were excluded after full‐text review, resulting in 12 studies being included in the systematic review. The study‐selection process is shown in Figure 1.

**Figure 1:**
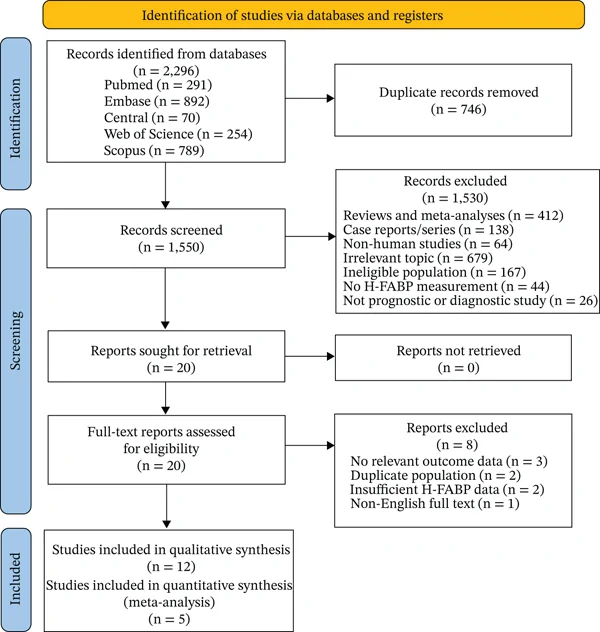
PRISMA 2020 flow diagram of study identification and selection.

Five studies provided extractable 2 × 2 prognostic event data and contributed to at least one quantitative synthesis [4, 13, 18–20]. The remaining seven studies were synthesised narratively, including studies reporting adjusted prognostic associations and studies primarily evaluating diagnostic or early risk‐stratification performance [11, 12, 14–17, 21].

The included studies covered heterogeneous clinical populations. Jeong et al. [11] studied 278 patients with elevated NT‐proBNP divided into ACS/cardiac arrest, non‐ACS cardiac disease and infection/sepsis groups. Banu et al. [12] evaluated 160 patients with confirmed STEMI. Matsumoto et al. [13] studied 1283 survivors of AMI during the convalescent phase. Garcia‐Valdecasas et al. [14] enrolled 165 patients presenting with acute chest pain and suspected AMI. Kurz et al. [15] evaluated 94 patients with suspected NSTE‐ACS. Viswanathan et al. [16] recruited 1080 patients with suspected ACS, of whom 955 were included in the final analysis after prespecified exclusions. McCann et al. [17] recruited 664 patients with ischaemic‐type chest pain; 555 fulfilled the analytic criteria and 1‐year follow‐up was available in 550. Kilcullen et al. [18] analysed 1448 patients with confirmed ACS. O′Donoghue et al. [4] analysed 2287 patients from the OPUS‐TIMI 16 ACS cohort. Suzuki et al. [19] evaluated 90 patients with ACS. Ishii et al. [20] studied 328 consecutive patients admitted with ACS within 6 h of chest‐pain onset. Nakata et al. [21] prospectively studied 133 patients presenting with suspected ACS.

Detailed study characteristics, including assay methodology, sampling time, H‐FABP thresholds, outcomes and renal‐function handling, are presented in Table 1.

### 3.2. Meta‐Analysed Outcomes

#### 3.2.1. Major Adverse Cardiovascular Events

Elevated H‐FABP was associated with higher odds of MACE. The pooled OR was 4.56 (95% CI 1.97–10.55; *p* < 0.001), corresponding to approximately 4.6‐fold higher odds of MACE among patients with elevated H‐FABP. Statistical heterogeneity was *I* ^2^ = 47*%*. Given the small number of studies contributing to this analysis, the heterogeneity estimate should be interpreted cautiously. The corresponding forest plot is shown in Figure 2.

**Figure 2:**
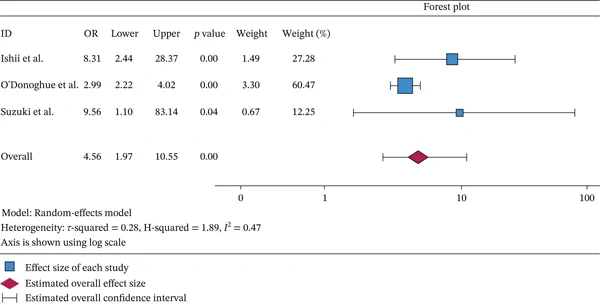
Forest plot of the association between elevated H‐FABP and major adverse cardiovascular events.

#### 3.2.2. All‐Cause Mortality

Elevated H‐FABP was associated with higher odds of all‐cause mortality. The pooled OR was 5.68 (95% CI 3.98–8.09; *p* < 0.001), corresponding to approximately 5.7‐fold higher odds of death among patients with elevated H‐FABP. Statistical heterogeneity was *I* ^2^ = 30*%*. Given the small number of contributing studies, this estimate should be interpreted cautiously. The forest plot is presented in Figure 3.

**Figure 3:**
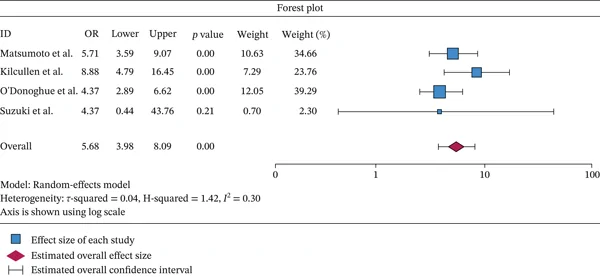
Forest plot of the association between elevated H‐FABP and all‐cause mortality.

#### 3.2.3. Heart Failure–Related Hospitalisation

Elevated H‐FABP was associated with higher odds of HF‐related hospitalisation, with a pooled OR of 4.29 (95% CI 2.94–6.26; *p* < 0.001). Statistical heterogeneity was *I* ^2^ = 0*%*; however, because only a small number of studies contributed to this analysis, this should not be interpreted as evidence of an absence of clinical heterogeneity. The forest plot is provided in Figure 4.

**Figure 4:**
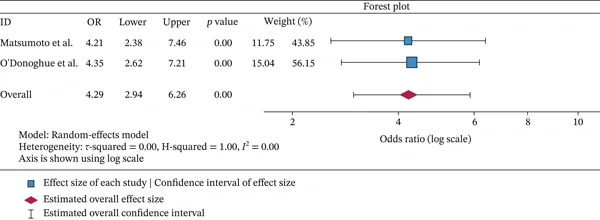
Forest plot of the association between elevated H‐FABP and heart failure–related hospitalisation.

#### 3.2.4. Recurrent Myocardial Infarction

Elevated H‐FABP was associated with higher odds of recurrent MI, with a pooled OR of 1.65 (95% CI 1.15–2.37; *p* = 0.01). Statistical heterogeneity was *I* ^2^ = 0*%*; however, because only a small number of studies contributed, this should not be interpreted as evidence of an absence of clinical heterogeneity. The forest plot is shown in Figure 5.

**Figure 5:**
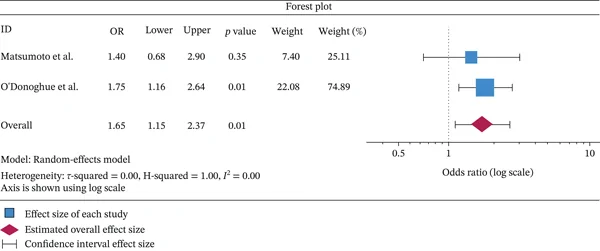
Forest plot of the association between elevated H‐FABP and recurrent myocardial infarction.

#### 3.2.5. Narratively Synthesised Prognostic Evidence

Garcia‐Valdecasas et al. [14] reported that elevated H‐FABP was independently associated with adverse events over 6 months in patients presenting with acute chest pain. The study‐specific composite included death, new MI occurring more than 24 h after admission, angina pectoris, congestive HF, revascularisation and other cardiac events. In the multivariable Cox model, elevated H‐FABP remained associated with outcome.

McCann et al. [17] evaluated patients presenting with acute ischaemic‐type chest pain and found that admission H‐FABP provided prognostic information for death or MI at 1 year. H‐FABP remained an independent predictor after adjustment for clinical characteristics, ECG findings, troponin, renal function and other biomarkers.

Banu et al. [12] reported higher mean H‐FABP levels among patients who died during the index hospitalisation than among survivors; however, this difference was not statistically significant. In contrast, hs‐cTnT was significantly associated with in‐hospital mortality.

Viswanathan et al. [16] analysed 955 patients with suspected ACS following prespecified exclusions. H‐FABP measured 12–24 h after symptom onset independently predicted death or readmission with MI over a median follow‐up of 18 months. Concentrations above 6.48 *μ*g/L were associated with adverse outcomes after multivariable adjustment, and H‐FABP retained prognostic information among troponin‐negative patients after adjustment for age and serum creatinine.

Kilcullen et al. [18] studied 1448 patients with confirmed ACS and found a graded association between increasing H‐FABP concentration and 12‐month all‐cause mortality. H‐FABP provided prognostic information in models incorporating GRACE clinical variables, troponin I, hs‐CRP and admission creatinine.

Jeong et al. [11] found that H‐FABP concentrations were associated with mortality in a mixed cohort of patients with elevated NT‐proBNP. An H‐FABP concentration ≥ 8.8 ng/mL was associated with an age‐adjusted hazard ratio of 3.25 for mortality compared with the reference category, and H‐FABP showed strong discrimination for short‐term mortality. Because the cohort included ACS/cardiac arrest, other cardiac disease and infection/sepsis groups, these findings should not be interpreted as deriving exclusively from an ACS population.

#### 3.2.6. Additional Diagnostic and Early Risk‐Stratification Evidence

Kurz et al. [15] evaluated 94 patients with suspected NSTE‐ACS and compared H‐FABP with high‐sensitivity troponin T, conventional troponin T and myoglobin. H‐FABP showed high sensitivity at presentation, but high‐sensitivity troponin T demonstrated at least comparable overall diagnostic performance at baseline and during serial sampling.

Nakata et al. [21] evaluated 133 patients presenting with suspected ACS. H‐FABP showed strong early diagnostic performance and had greater sensitivity than the conventional biomarkers studied for identifying patients requiring emergency hospitalisation, coronary angiography or intervention within 1 days, particularly when measured within 6 h of symptom onset.

## 4. Discussion

This systematic review and meta‐analysis found that elevated H‐FABP was associated with higher odds of MACE, all‐cause mortality, HF‐related hospitalisation and recurrent MI in patients with confirmed or suspected ACS. These pooled findings demonstrate prognostic associations but should not be interpreted as establishing independent prognostic value because the quantitative estimates were derived from unadjusted event data. Several individual studies reported associations after multivariable adjustment; however, the available adjusted estimates could not be quantitatively pooled because of heterogeneity in covariate adjustment, effect measures and outcome reporting.

Compared with the 2017 systematic review by Jones et al. [7], which synthesised evidence available to mid‐2015 without formal meta‐analysis, the present review provides an updated quantitative synthesis. Five of the 12 included studies contributed extractable data to at least one pooled analysis, whereas seven were synthesised narratively. The present review also evaluated HF‐related hospitalisation and recurrent MI in addition to mortality and MACE. Nevertheless, the small number of studies contributing to each pooled outcome requires cautious interpretation of both the magnitude and consistency of the observed associations.

The quantitative findings are broadly compatible with several individual observational studies reporting adjusted estimates. Viswanathan et al. [16] found that H‐FABP remained associated with death or readmission with MI after multivariable adjustment and provided prognostic information among patients who were troponin‐negative. Kilcullen et al. [18] similarly reported a graded association between H‐FABP and 12‐month mortality after adjustment for GRACE clinical variables, troponin, hs‐CRP and other factors. O′Donoghue et al. [4] found that H‐FABP remained associated with the composite of death, MI or HF and with death and HF individually after adjustment for demographics, clinical characteristics, creatinine clearance, index diagnosis, ECG findings and troponin I. McCann et al. [17] also demonstrated independent prognostic information from admission H‐FABP in patients presenting with ischaemic‐type chest pain.

These adjusted individual‐study findings are important because the pooled estimates in the present meta‐analysis were based on crude 2 × 2 event data. The pooled ORs therefore describe associations between H‐FABP status and outcome but do not establish that H‐FABP is independently associated with prognosis after adjustment for established risk factors.

Importantly, evidence of prognostic association is distinct from evidence of clinically meaningful incremental prediction. Kilcullen et al. [18] reported that H‐FABP provided information additional to GRACE variables, troponin and hs‐CRP, whereas O′Donoghue et al. [4] reported complementary information from H‐FABP, troponin I and BNP. However, these observations derive from individual cohorts. No pooled evidence was available demonstrating that addition of H‐FABP to established ACS risk models improves discrimination, calibration, reclassification or clinical decision‐making.

Measures such as change in the C‐statistic, net reclassification improvement, integrated discrimination improvement and decision‐curve benefit could not be quantitatively synthesised. Whether H‐FABP meaningfully improves prognostic assessment beyond high‐sensitivity cardiac troponin, GRACE, TIMI, ECG findings, renal function and natriuretic peptides therefore remains uncertain

This distinction is particularly relevant in contemporary guideline‐directed ACS management. The 2023 European Society of Cardiology guideline emphasises clinical assessment, ECG findings, serial high‐sensitivity cardiac troponin testing and established clinical risk assessment in the diagnosis and management of ACS [22]. H‐FABP is not currently incorporated into routine guideline‐endorsed prognostic pathways. The findings of the present review therefore do not establish a basis for replacing or routinely adding H‐FABP to established ACS assessment pathways.

Substantial heterogeneity was present in H‐FABP measurement. The included studies used immunoturbidimetric assays, ELISA‐based methods, chemiluminescent immunometric assays, sequential sandwich immunoassays and rapid qualitative testing. Thresholds used to define H‐FABP elevation also varied considerably. Some were based on reference‐population percentiles, others on assay detection limits, ROC‐derived thresholds, study medians or qualitative test positivity. These differences complicate comparison between studies and prevent identification of a single clinically validated H‐FABP threshold.

Timing of measurement also varied substantially. H‐FABP was assessed at emergency presentation in several studies [14, 17, 19–21], 12–24 h after symptom onset in others [16, 18], approximately 41 h after symptom onset in O′Donoghue et al. [4] and at a median of 20 days after AMI in Matsumoto et al. [13]. Because H‐FABP concentrations change rapidly following myocardial injury, measurements obtained at these different phases do not necessarily represent biologically equivalent exposures. The pooled category of “elevated H‐FABP” should therefore not be interpreted as a standardised biomarker measurement.

Clinical heterogeneity was similarly considerable. Included populations ranged from confirmed STEMI [12], confirmed mixed ACS [4, 18–20] and post‐AMI survivors [13] to broader suspected‐ACS or chest‐pain populations [14–17, 21]. In suspected‐ACS cohorts, not all recruited participants necessarily received a final diagnosis of ACS. Consequently, the magnitude and interpretation of the association between H‐FABP and outcome may differ according to ACS phenotype and diagnostic certainty.

Outcome definitions also varied. MACE did not represent a uniform endpoint and included differing combinations of mortality, MI, recurrent ACS, angina, heart failure, revascularisation and other cardiovascular events. Garcia‐Valdecasas et al. [14], for example, included death, new MI, angina, congestive HF, revascularisation and other cardiac events, whereas Suzuki et al. [19] defined 30‐day adverse events as cardiac death or recurrent ACS. O′Donoghue et al. [4] evaluated death, recurrent MI and congestive HF individually and as a composite. Pooling study‐specific composite outcomes therefore provides an overall measure of association but not an estimate for one uniformly defined clinical endpoint.

Follow‐up duration ranged from in‐hospital outcomes [12] and 30‐day events [19] to 6‐month [14, 20], 10‐month [4], 12‐month [17, 18], median 18‐month [16] and approximately 5‐year follow‐up [13]. Binary ORs at these different time horizons are not directly equivalent because cumulative event opportunities differ. Time‐to‐event estimates would be preferable, but consistent adjusted hazard ratios with usable variance estimates were not available across studies.

Renal function represents a particularly important consideration. H‐FABP is a low‐molecular‐weight protein whose circulating concentration is influenced by renal elimination [6]. Renal impairment is also independently associated with adverse cardiovascular outcomes, creating potential confounding. The included studies handled renal function inconsistently. Kurz et al. [15] excluded patients with a glomerular filtration rate < 60 mL/min/1.73 m^2^. O′Donoghue et al. [4] excluded substantial renal impairment and adjusted for creatinine clearance. Ishii et al. [20] excluded patients above a predefined serum‐creatinine threshold and incorporated creatinine into multivariable analyses. Viswanathan et al. [16] adjusted analyses for serum creatinine. Kilcullen et al. [18] incorporated admission creatinine through the GRACE variables, and Matsumoto et al. [13] explicitly adjusted for chronic kidney disease defined using eGFR. In contrast, renal adjustment was limited or not clearly reported in several other studies.

This is clinically important because elevated H‐FABP in renal dysfunction may partly reflect impaired clearance rather than myocardial injury alone. Garcia‐Valdecasas et al. [14] observed elevated H‐FABP in a large proportion of non‐AMI patients with renal failure, whereas Matsumoto et al. [13] found that reduced eGFR was strongly associated with elevated H‐FABP concentrations. Residual confounding by renal function therefore remains a plausible contributor to at least part of the observed prognostic association.

More broadly, H‐FABP forms part of continuing research into multimarker prognostic assessment in ACS. Recent evidence also demonstrates interest in biomarkers reflecting alternative biological pathways. Karakayali et al. [8], for example, studied 556 patients with ACS and found that admission malondialdehyde, a marker of oxidative stress, remained independently associated with in‐hospital mortality after multivariable analysis. Such findings do not imply that malondialdehyde or other emerging biomarkers should replace established markers. Rather, they illustrate the need for future biomarker research to demonstrate genuine incremental information beyond established clinical predictors rather than merely statistically significant associations with outcome.

## 5. Limitations

Several limitations should be considered when interpreting the findings. First, only five studies contributed to the quantitative synthesis and only two to four studies contributed to each individual pooled outcome. Estimates of between‐study heterogeneity, including *I* ^2^ values of 0% in some analyses, are imprecise with so few studies and should not be interpreted as demonstrating an absence of underlying clinical or methodological heterogeneity. The small evidence base also prevented reliable subgroup meta‐analysis, leave‐one‐out analysis or sensitivity analysis according to ACS phenotype, H‐FABP assay, sampling time, threshold, troponin status, renal function or risk of bias. Second, formal assessment of publication bias using funnel‐plot asymmetry or regression‐based tests such as Egger′s test was not undertaken because fewer than 10 studies contributed to each pooled outcome. Such tests would be unreliable in this setting. Nevertheless, publication bias cannot be excluded. Preferential publication of positive biomarker studies, selective reporting of favourable outcomes and broader small‐study effects may have contributed to overestimation of the observed associations. Third, the pooled ORs were calculated from unadjusted 2 × 2 event data. They therefore do not account for potentially important confounding by age, renal function, troponin status, ACS severity, ventricular function, cardiovascular comorbidity or other established prognostic factors. This concern is supported by the QUIPS assessment, in which several studies were judged to have moderate or high risk of bias in the confounding domain. Adjusted findings from individual studies are more informative regarding independent prognostic association but could not be pooled because of differing covariate sets and effect measures. Fourth, substantial clinical and methodological heterogeneity remained despite relatively modest statistical heterogeneity in some analyses. Included populations ranged from confirmed STEMI and NSTE‐ACS to mixed and suspected‐ACS cohorts. Assay platforms, units, thresholds, sampling times, follow‐up periods and outcome definitions also differed. These variations limit the extent to which one pooled effect estimate can be applied to a specific ACS phenotype or clinical setting. Fifth, renal function was inconsistently handled across studies. Because impaired renal clearance can elevate H‐FABP independently of myocardial injury and renal dysfunction itself confers adverse prognosis, residual confounding by kidney function may partly account for the observed associations. The available evidence does not permit reliable estimation of H‐FABP′s prognostic performance specifically across different stages of chronic kidney disease. Sixth, variation in follow‐up duration limits interpretation of pooled binary ORs. Time‐to‐event analyses would more appropriately account for differing periods at risk; however, sufficiently comparable adjusted hazard ratios were unavailable for quantitative synthesis. Seventh, the review included several studies primarily focused on diagnostic or early risk‐stratification performance because such studies were eligible under the registered protocol. These studies were retained for narrative context and did not contribute to pooled prognostic estimates unless suitable clinical event data were available. The distinction between diagnostic accuracy, early clinical triage and long‐term prognostic value should therefore be maintained when interpreting the overall evidence base. Eighth, only English‐language full‐text reports were eligible, introducing the possibility of language bias. Although five major bibliographic databases were searched and reference‐list screening was undertaken, unpublished studies and poorly indexed reports may still have been missed. Finally, the available evidence was insufficient to establish incremental predictive value or clinical utility. This review could not determine whether adding H‐FABP to high‐sensitivity troponin, GRACE, TIMI, ECG findings, renal function, natriuretic peptides or other established predictors improves discrimination, calibration, reclassification, clinical decision‐making or patient outcomes. Statistically significant associations should therefore not be interpreted as evidence that routine H‐FABP testing improves ACS management.

Future prospective studies should use standardised H‐FABP assays, prespecified thresholds and sampling times, consistent clinical outcome definitions and adequate adjustment for renal function and other established prognostic factors. Studies should report adjusted time‐to‐event estimates and formally evaluate incremental predictive performance, including discrimination, calibration, reclassification and decision‐curve analysis. Ultimately, prospective evidence is required to determine whether incorporation of H‐FABP into multimarker or established clinical risk models results in clinically meaningful improvements in management or outcomes.

## 6. Conclusion

This systematic review and meta‐analysis found that elevated H‐FABP is associated with higher odds of all‐cause mortality, MACE, HF‐related hospitalisation and recurrent MI in patients with ACS. However, the pooled associations were derived from unadjusted estimates from a small number of studies and should not be interpreted as establishing independent prognostic value. H‐FABP may warrant further evaluation as a prognostic biomarker, but its incremental value beyond established clinical risk scores, high‐sensitivity cardiac troponin, renal function and other established clinical and biochemical risk markers remains uncertain. Large prospective studies using standardised H‐FABP assays and thresholds and formally evaluating discrimination, calibration, reclassification and clinical utility are required before H‐FABP can be incorporated into routine ACS risk‐stratification pathways.

## Author Contributions

Ahmed Elmewafy and Eyad Jamileh collected and analysed data, contributed to drafting and revised the manuscript. Abdullah Hussain and Loay Kamel contributed to data collection and drafting of the manuscript. Omar Farooq contributed to revision of the manuscript and critical review for important intellectual content. Hany Gamal contributed to revision of the manuscript and critical review for important intellectual content. Ibrahim Antoun contributed to study design, supervision, critical revision and final approval.

## Funding

No funding was received for this manuscript.

## Disclosure

All authors reviewed and approved the final manuscript. This systematic review was registered in the PROSPERO International Register of Systematic Reviews. Registration Number: CRD420251113979.

## Ethics Statement

A statement of ethics is not applicable because this study is based exclusively on previously published literature.

## Conflicts of Interest

The authors declare no conflicts of interest.

## Supporting Information

Additional supporting information can be found online in the Supporting Information section.

## Supporting information


**Supporting Information 1** File S1: PRISMA 2020 checklist.

**Supporting Information 2** File S2: Complete database‐specific search strategies for PubMed, Embase, CENTRAL, Web of Science and Scopus, including search dates, search strings and database‐specific retrieval counts.

**Supporting Information 3** Figure S1: Visual summary of domain‐level QUIPS risk‐of‐bias assessments across included studies.

## Data Availability

The data that support the findings of this study are available from the corresponding author upon reasonable request.
