# Supplementary material for: Prognostic Value of Heart‐Type Fatty Acid‐Binding Protein in Patients With Acute Coronary Syndrome: A Systematic Review and Meta‐Analysis
Source: Cardiovasc Ther. 2026 Oct 20;2026:4836688. doi: 10.1155/cdr/4836688 (PMC13681350; doi:10.1155/cdr/4836688)
Supplement: Supplementary file 1 [file CDR-2026-4836688-s001.docx]

**Supplementary File S2. Complete Electronic Search Strategies**

**Search coverage.** PubMed, Embase, the Cochrane Central Register of Controlled Trials (CENTRAL), Web of Science Core Collection, and Scopus were searched from database inception to 30 August 2026. No publication-date restriction was applied at either the database-search or study-screening stage.

**Search summary**

| **Database** | **Final search date** | **Records retrieved** |
| --- | --- | --- |
| PubMed | 30 August 2026 | 291 |
| Embase | 30 August 2026 | 892 |
| CENTRAL | 30 August 2026 | 70 |
| Web of Science Core Collection | 30 August 2026 | 254 |
| Scopus | 30 August 2026 | 789 |
| **Total** |  | **2,296** |

**PubMed (291 records; final search 30 August 2026)**

(

"heart-type fatty acid binding protein"[Title/Abstract] OR

"fatty acid binding protein"[Title/Abstract] OR

"fatty acid-binding protein"[Title/Abstract] OR

"FABP"[Title/Abstract] OR

"FABP3"[Title/Abstract] OR

"H-FABP"[Title/Abstract]

)

AND

(

"acute coronary syndrome"[Title/Abstract] OR

"ACS"[Title/Abstract] OR

"myocardial infarction"[Title/Abstract] OR

"MI"[Title/Abstract] OR

"STEMI"[Title/Abstract] OR

"NSTEMI"[Title/Abstract] OR

"unstable angina"[Title/Abstract] OR

"coronary artery disease"[Title/Abstract] OR

"coronary disease"[Title/Abstract] OR

"ischemic heart disease"[Title/Abstract]

)

AND

(

"prognosis"[Title/Abstract] OR

"prognostic"[Title/Abstract] OR

"prediction"[Title/Abstract] OR

"predictive value"[Title/Abstract] OR

"risk"[Title/Abstract] OR

"mortality"[Title/Abstract] OR

"outcomes"[Title/Abstract] OR

"MACE"[Title/Abstract] OR

"readmission"[Title/Abstract] OR

"follow-up"[Title/Abstract] OR

"cardiovascular events"[Title/Abstract] OR

"clinical outcome"[Title/Abstract]

)

**Embase (892 records; final search 30 August 2026)**

(

'heart type fatty acid binding protein':ti,ab,kw OR

'heart-type fatty acid binding protein':ti,ab,kw OR

'fatty acid binding protein':ti,ab,kw OR

'fatty acid-binding protein':ti,ab,kw OR

FABP:ti,ab,kw OR

FABP3:ti,ab,kw OR

'H-FABP':ti,ab,kw OR

'heart fatty acid binding protein'/exp

)

AND

(

'acute coronary syndrome':ti,ab,kw OR

ACS:ti,ab,kw OR

'myocardial infarction':ti,ab,kw OR

MI:ti,ab,kw OR

STEMI:ti,ab,kw OR

NSTEMI:ti,ab,kw OR

'unstable angina':ti,ab,kw OR

'coronary artery disease':ti,ab,kw OR

'coronary disease':ti,ab,kw OR

'ischemic heart disease':ti,ab,kw OR

'ischaemic heart disease':ti,ab,kw OR

'acute coronary syndrome'/exp OR

'myocardial infarction'/exp

)

AND

(

prognosis:ti,ab,kw OR

prognostic:ti,ab,kw OR

prediction:ti,ab,kw OR

'predictive value':ti,ab,kw OR

risk:ti,ab,kw OR

mortality:ti,ab,kw OR

outcome*:ti,ab,kw OR

MACE:ti,ab,kw OR

readmission*:ti,ab,kw OR

'follow-up':ti,ab,kw OR

'follow up':ti,ab,kw OR

'cardiovascular event*':ti,ab,kw OR

'clinical outcome*':ti,ab,kw OR

'prognosis'/exp OR

'mortality'/exp

)

**Cochrane Central Register of Controlled Trials (CENTRAL) (70 records; final search 30 August 2026)**

("heart type fatty acid binding protein" OR "fatty acid binding protein" OR FABP OR FABP3 OR HFABP)

AND

("acute coronary syndrome" OR ACS OR "myocardial infarction" OR MI OR STEMI OR NSTEMI OR "unstable angina" OR "coronary artery disease" OR "coronary disease" OR "ischemic heart disease" OR "ischaemic heart disease")

AND

(prognosis OR prognostic OR prediction OR "predictive value" OR risk OR mortality OR outcomes OR MACE OR readmission OR "follow up" OR "cardiovascular events" OR "clinical outcome")

**Web of Science Core Collection (254 records; final search 30 August 2026)**

TS=(

"heart-type fatty acid binding protein" OR

"heart type fatty acid binding protein" OR

"fatty acid binding protein" OR

"fatty acid-binding protein" OR

FABP OR FABP3 OR "H-FABP"

)

AND

TS=(

"acute coronary syndrome" OR ACS OR

"myocardial infarction" OR MI OR STEMI OR NSTEMI OR

"unstable angina" OR "coronary artery disease" OR

"coronary disease" OR "ischemic heart disease" OR

"ischaemic heart disease"

)

AND

TS=(

prognos* OR predict* OR risk OR mortalit* OR outcome* OR

MACE OR readmission* OR "follow-up" OR "follow up" OR

"cardiovascular event*" OR "clinical outcome*"

)

**Scopus (789 records; final search 30 August 2026)**

TITLE-ABS-KEY(("heart-type fatty acid binding protein" OR "fatty acid binding protein" OR "fatty acid-binding protein" OR FABP OR FABP3 OR H-FABP))

AND

TITLE-ABS-KEY(("acute coronary syndrome" OR ACS OR "myocardial infarction" OR MI OR STEMI OR NSTEMI OR "unstable angina" OR "coronary artery disease" OR "coronary disease" OR "ischemic heart disease" OR "ischaemic heart disease"))

AND

TITLE-ABS-KEY((prognos* OR predict* OR risk OR mortalit* OR outcome* OR MACE OR readmission* OR "follow-up" OR "cardiovascular event*" OR "clinical outcome*"))
